# MICA: desktop software for comprehensive searching of DNA databases

**DOI:** 10.1186/1471-2105-7-427

**Published:** 2006-10-03

**Authors:** William A Stokes, Benjamin S Glick

**Affiliations:** 1 GSL Biotech, LLC, 5211 S. Kenwood Ave. Chicago, IL 60615, USA; 2 Department of Molecular Genetics and Cell Biology, and Institute for Biophysical Dynamics, University of Chicago, 920 East 58th Street, Chicago, IL 60637, USA

## Abstract

**Background:**

Molecular biologists work with DNA databases that often include entire genomes. A common requirement is to search a DNA database to find exact matches for a nondegenerate or partially degenerate query. The software programs available for such purposes are normally designed to run on remote servers, but an appealing alternative is to work with DNA databases stored on local computers. We describe a desktop software program termed MICA (*K*-Mer Indexing with Compact Arrays) that allows large DNA databases to be searched efficiently using very little memory.

**Results:**

MICA rapidly indexes a DNA database. On a Macintosh G5 computer, the complete human genome could be indexed in about 5 minutes. The indexing algorithm recognizes all 15 characters of the DNA alphabet and fully captures the information in any DNA sequence, yet for a typical sequence of length *L*, the index occupies only about 2*L *bytes. The index can be searched to return a complete list of exact matches for a nondegenerate or partially degenerate query of any length. A typical search of a long DNA sequence involves reading only a small fraction of the index into memory. As a result, searches are fast even when the available RAM is limited.

**Conclusion:**

MICA is suitable as a search engine for desktop DNA analysis software.

## Background

Researchers are increasingly working with large DNA databases. For example, the human genome is approximately 3 gigabases. Searching these databases has traditionally been done by using web applications to communicate with dedicated servers. As an alternative analysis tool, desktop computers offer richer and more responsive graphical interfaces. Desktop software programs are available for displaying and manipulating plasmids and other relatively small DNA molecules. Such functionality could theoretically be extended to large DNA databases, because typical desktop computers now have hard disk capacities of hundreds of GB. However, most bioinformatics applications load all of the relevant DNA data into main memory, so the RAM capacity of desktop computers remains a limitation. The challenge is to create desktop DNA analysis software that accomodates large DNA databases while using modest amounts of RAM.

A basic requirement for such software is rapid searching of a DNA database to find all exact matches for a query sequence. The desired search speeds can only be achieved by indexing the database. One well-characterized indexing strategy is to generate a suffix tree [1]. Although suffix trees have been used productively for some molecular biology applications, such as aligning whole genomes [2], they consume large amounts of memory, up to 15 bytes or more per base. Suffix arrays are more compact than suffix trees and can provide similar search capabilities, but they still require 4–8 bytes per base [3]. These conventional suffix-based search strategies rely on loading the entire index into memory, so they are not currently suitable for desktop analysis of large DNA databases. A potential solution to this problem is to generate a compressed suffix array using a Burrows-Wheeler transform [4]. The indexes generated by this method occupy as little as 0.3 bytes per base, but indexing is slow: generation of a compressed suffix array for the human genome required many hours on a desktop computer [4].

Non-suffix-based indexing stategies are currently in more widespread use for DNA databases. The SSAHA algorithm divides a DNA sequence into nonoverlapping *K*-mers, and stores the position of these *K*-mers in a hash table [5]. A similar indexing method is used by the BLAT algorithm [6]. Both SSAHA and BLAT generate relatively small indexes, on the order of 1 byte or less per base, and they can be orders of magnitude faster than FASTA or BLAST, which index the query sequence rather than the database [7-9]. SSAHA and BLAT have proven to be powerful for applications such as mapping sequence reads to a genome, or aligning mRNA sequences with the corresponding genomic DNA sequences. However, SSAHA and BLAT have limitations. Unlike suffix-based algorithms, which can identify all matches to any query sequence, SSAHA cannot detect a match of fewer than *K *bases, and requires 2*K*-1 consecutive matching bases to guarantee that a match will be registered. Because SSAHA sorts the search results, efficient searching is achieved by ignoring the *K*-mers that occur most frequently in the database. Similarly, BLAT sacrifices completeness for speed.

These various algorithms have been designed with the assumption that the complete index of a DNA database will be stored in main memory. Such algorithms are inconvenient for desktop applications. An index might be too large to fit within the RAM of a personal computer. Even if sufficient RAM were available, loading an index might be time-consuming. For example, a compressed suffix array of the human genome occupies about 1 GB [4], and reading those data from a hard disk into RAM would require tens of seconds, an unacceptable delay for users who expect fast access to information. We are developing software that will allow users to open and browse a large DNA database as rapidly as they open a plasmid file. The main innovation of our approach is to leave the index on disk, and to retrieve the relevant data selectively during a search.

For general purpose DNA analysis software, the database searches need to be comprehensive. Applications include searching whole chromosomes to create complete lists of restriction sites or oligonucleotide hybridization sites. We have met this need with an indexing and searching algorithm called MICA (*K*-Mer Indexing with Compact Arrays). The indexes occupy ~2 bytes per base and can be generated quickly. Unlike most other algorithms, MICA indexes not only the standard nucleotide base characters A, C, G, and T, but also the degenerate base characters B, D, H, K, M, R, S, V, W, Y, and N. Efficient search procedures identify all matches for a nondegenerate or partially degenerate query of any length. When a file is being searched, only a fraction of the index is loaded into memory. The result is that desktop computers with modest amounts of RAM can rapidly open and search large DNA databases.

## Implementation

### Index structure

Indexing a DNA sequence can be achieved by scanning the sequence with a window of width *K*. For a sequence of length *L*, sliding the window one base at a time yields *L *- *K *+ 1 overlapping *K*-mers. A MICA index uses arrays to store all of the positions for each *K*-mer in the subject DNA sequence. Because chromosomal DNA molecules can be up to several hundred million bases long, the *K*-mer position values would normally be represented as 4-byte integers. We reduce this data storage requirement by dividing the sequence into *C *separate "chunks" of 65,535 (2^16^-1) bases, where *C *= ceiling(*L*/65,535). The intra-chunk positions of each nondegenerate *K*-mer are stored as 2-byte integers. The absolute positions of a *K*-mer within the full DNA sequence can then be calculated with the aid of a list specifying the number of instances of the *K*-mer within each chunk.

In addition to the four nondegenerate base characters, there are 10 characters (B, D, H, K, M, R, S, V, W, and Y) representing partially degenerate base possibilities, plus one character (N) that represents any possible base. Partially degenerate *K*-mers must be recognized if the index is to capture all of the information in any DNA sequence. It would be wasteful to create an array for all of the different partially degenerate *K*-mers because most of the array elements would typically be empty. Instead, for each partially degenerate *K*-mer that is actually present in a subject sequence, we use a 4-byte integer to record the absolute position of the *K*-mer, followed by a *K*-byte string to record the sequence of the *K*-mer. This approach is inefficient with regard to data storage, but most DNA sequences contain very few partially degenerate *K*-mers, and the simplicity of the data format facilitates searching. A separate strategy is used for stretches of N's, which are commonly used to indicate undefined portions of a sequence. Here *S *designates the number of "N-stretches" of *K *or more consecutive N's. MICA efficiently indexes N-stretches by recording their starting positions and their lengths.

Also stored in the index is the topology of the subject DNA molecule (linear or circular). With circular DNA molecules, MICA finds matching sequences that span the numerically defined origin.

Table 1 summarizes the MICA file structure, together with the generic data storage requirements for each part of the file. As an example, Table 1 lists the specific data storage requirements for human chromosome 1, which at ~246 million bases represents one of the longest DNA molecules that needs to be indexed. To ensure that memory addresses can be represented as 4-byte integers, we have constrained MICA to index individual DNA sequences of no more than 1 gigabase.

**Table 1:** MICA file structure and data storage requirements

File Element	Generic Storage Requirement (bytes)	Storage Requirement for Chromosome 1 (bytes)
**Sequence Segment**		
**A. **Segment Format	1	1
**B. **Segment Size	4	4
**C. **Sequence Properties	1	1
**D. **DNA Sequence	*L*	245,522,847 (234 MB)
SEGMENT TOTAL	6 + *L*	245,522,853 (234 MB)
**Index Segment**		
**E. **Segment Format	1	1
**F. **Segment Size	4	4
**G. **Index Properties	1	1
**H. **Chunk Counts Summary	4^*K*+1^	*K *= 4: 1,024 (1 KB) *K *= 6: 16,384 (16 KB)
**I. **Degenerate *K*-mer Count	4	4
**J. **N-Stretch Count (*S*)	4	4
**K. **Chunk Data Array	(4^K ^* *C *+ number of nondegenerate *K*-mers) * 2	*K *= 4: 447,573,936 (427 MB) *K *= 6: 476,350,748 (454 MB)
**L. **Degenerate Data Array	(number of partially degenerate *K*-mers) * (4 + *K*)	*K *= 4: 1,752 (1.7 KB) *K *= 6: 3,650 (3.6 KB)
**M. **N-Stretch Data Array	8*S*	296
SEGMENT TOTAL	Typically about 2*L *bytes.	*K *= 4: 447,577,022 (427 MB) *K *= 6: 476,371,092 (454 MB)

A MICA file consists of a Sequence Segment (elements A – D) followed by an Index Segment (elements E – M). If a sequence occupies more than 16 chunks, then loading of a MICA index consists of reading elements A – C of the Sequence Segment, skipping over the DNA sequence, and reading elements E – J of the Index Segment. The parameters for human chromosome 1 were: *L *= 245,522,847 bases; *C *= 3,747 chunks; *S *= 37 N-stretches (total of 22,695,000 N's).

(A) A single byte is used to specify the Sequence Segment format.

(B) The size of the Sequence Segment is specified by a 4-byte integer.

(C) A single byte is used to record properties of the sequence, including its topology (linear or circular) and its strandedness (single- or double-stranded).

(D) The DNA sequence is stored as a string of uppercase ASCII characters.

(E) A single byte is used to specify the Index Segment Format.

(F) The size of the Index Segment is specified by a 4-byte integer.

(G) A single byte is used to record the byte order ("endianness") of the index. If the byte order is opposite to that of the machine being used to run the queries, MICA corrects the byte order when processing the index data.

(H) The Chunk Counts Summary is a list of 4^*K *^4-byte integers representing the total number of times each nondegenerate *K*-mer appears in the sequence. For the MICA index, the 4-base nondegenerate DNA alphabet is arranged in the order G, A, T, C. Thus, the first nondegenerate *K*-mer listed is GGGG (*K *= 4) or GGGGGG (*K *= 6), and the last one listed is CCCC (*K *= 4) or CCCCCC (*K *= 6). This lexicographical order yields contiguous index reads for *K*-mers that end in the most common partially degenerate bases: R (A or G), Y (C or T), and W (A or T).

(I) The Degenerate *K*-mer Count is a 4-byte integer representing the total number of partially degenerate *K*-mers in the sequence.

(J) The N-Stretch Count *S *is a 4-byte integer representing the number of separate stretches of *K *or more consecutive N's.

(K) The Chunk Data Array is divided into 4^*K *^partitions corresponding to the 4^*K *^nondegenerate *K*-mers. Each partition contains a list of 2-byte integers representing the number of times the *K*-mer is present in each of the *C *chunks, followed by a list of 2-byte integers representing the intra-chunk positions of the *K*-mer in each of the *C *chunks. The first partition contains the data for GGGG (*K *= 4) or GGGGGG (*K*= 6), and the second partition contains the data for GGGA (*K *= 4) or GGGGGA (*K *= 6), and so on.

(L) The Degenerate Data Array is a list of the partially degenerate *K*-mers. Each partially degenerate *K*-mer is represented as a 4-byte integer that marks the absolute position of the *K*-mer, followed by a *K*-byte string that encodes the sequence of the *K*-mer.

(M) The N-Stretch Data Array consists of *S *pairs of 4-byte integers that represent the starting positions and lengths of the N-stretches.

### Choice of *K*

The main body of a MICA index is the Chunk Data Array, which stores the positions of the nondegenerate *K*-mers (Table 1). The total number of position values is largely independent of *K*. However, there are 4^*K *^different nondegenerate *K*-mers, so the Chunk Data Array is divided into 4^*K *^partitions. Each partition is divided into *C *sub-partitions that contain the intra-chunk position values. The sizes of these sub-partitions are recorded in a list at the beginning of the partition. As a result, the practical upper limit for *K *is 7, because for *K *= 8 there would be 65,536 sub-partitions per chunk, and the lists of sub-partition sizes would occupy more space than the lists of *K*-mer position values. The practical lower limit for *K *is 3, because reducing *K *increases the size of the partitions that must be read from disk, thereby slowing most searches.

### Index creation

To create a MICA index, a subject DNA sequence in FASTA format [10] is scanned using a window of width *K*. Both uppercase and lowercase characters are recognized. An initial scan fills in the data for index elements E – J (Table 1). Then the appropriate memory for the data arrays (index elements K – M) is allocated, and a second scan fills in the positions of the *K*-mers and N-stretches. These operations are fast, partly because building the index requires no sorting.

If sufficient memory is available, indexing speed is maximized by building the entire index in memory and then writing to disk in a single step. In the case of human chromosome 1, this process requires about 0.67 GB of RAM, an amount that is available on many desktop computers. If memory is limiting, only a subset of the *K*-mer position values are stored in memory at a given time, and the index is written to disk in multiple steps.

Currently, MICA embeds a copy of the DNA sequence within the file. This sequence consists of uppercase characters in 8-bit ASCII format, and therefore occupies *L *bytes. The original sequence file is then dispensable for searching. For future applications, MICA will be integrated with software that automatically generates a suitably formatted DNA sequence.

### General search strategy

If a DNA sequence occupies more than 16 chunks (~1 megabase), only elements A – C and E – J of the MICA file are initially read from disk (Table 1). These reads are very fast because they involve a small amount of data, just over 1 KB for *K *= 4 or just over 16 KB for *K *= 6. During a search, MICA uses the data from this first portion of the index to find the relevant position values. For example, to find the positions of a nondegenerate *K*-mer, an entry in the Chunk Counts Summary (index element H) indicates where the relevant position values can be read from the Chunk Data Array (index element K). Thus, MICA selectively reads only the essential data from disk, thereby performing efficient I/O operations and minimizing RAM usage.

**Figure 1:**
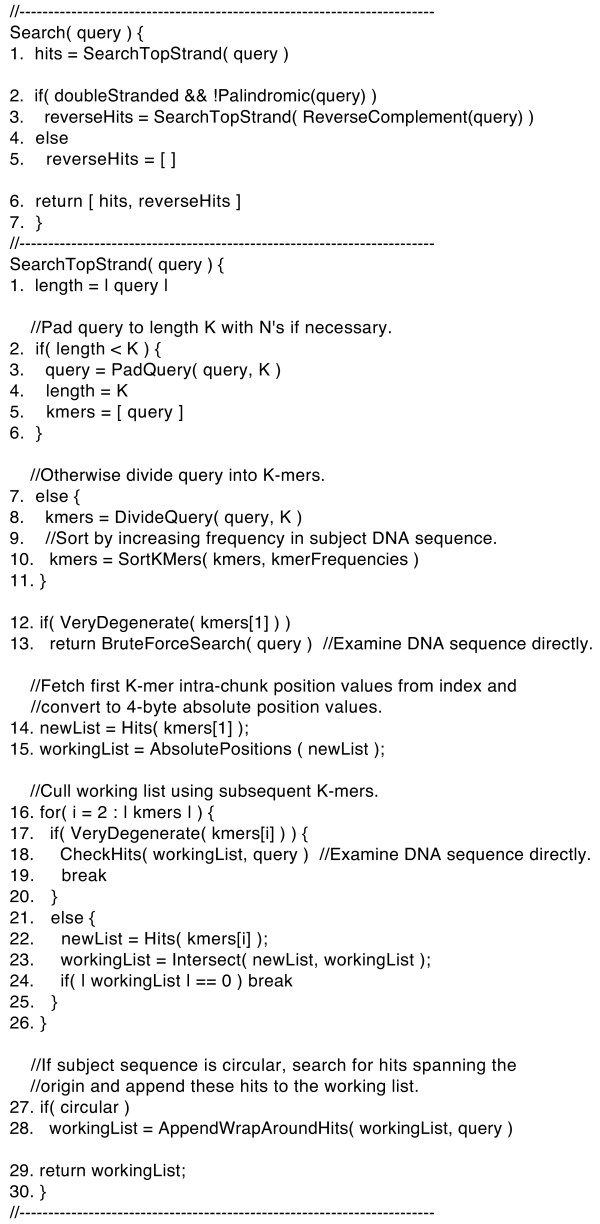
**Pseudocode summary of the basic MICA search routines**. See the text for additional information about memory management, intersection algorithms, and merge operations for degenerate *K*-mers.

Figure 1 provides a pseudocode summary of the basic MICA search routines. The query length *Q *can range from one base to the length of the subject DNA sequence. Both strands of the DNA molecule are searched. For a query that is palindromic–i.e., identical to its reverse complement–a single search is performed. For a query that is nonpalindromic, two successive searches are performed, one with the query and another with the reverse complement of the query. If the DNA molecule is circular, the initial search is followed by a secondary search for matches that span the origin. The key step in this secondary search involves dividing the query in half and then checking for one of two possibilities: either the first half-query matches within the last *Q*-1 bases of the DNA sequence, or the second half-query matches within the first *Q*-1 bases of the DNA sequence.

If a query is shorter than *K *bases, it is extended to *K *bases by adding N's, and is then treated as being partially degenerate (see below). If a query is exactly *K *bases, the search consists of converting the 2-byte intra-chunk position values for that *K*-mer to 4-byte absolute position values. If a query is longer than *K *bases, it is decomposed into constituent *K*-mers, which are examined as follows. The list of intra-chunk position values for the first *K*-mer is read from the index and converted to absolute position values, thereby creating an initial working list. Each value in the working list is then compared with the second *K*-mer list. The result is a new working list, which indicates where both the first and second *K*-mers from the query match the subject DNA sequence. This new working list is then compared with the next *K*-mer list, and so on. In this manner, MICA progressively trims the initial working list to generate a final list of matches.

With a query longer than *K *bases, the constituent *K*-mers are examined in increasing order of their frequency of appearance in the subject DNA sequence. For example, a search for the 12-mer AAAACCCCGGGG using *K *= 4 might involve calculating the positions for CCCC, then comparing each CCCC position against the list of positions for GGGG, then comparing each CCCCGGGG position against the list of positions for AAAA, which in this case would be the most common of the three 4-mers. This strategy of starting with the rarest *K*-mer can significantly accelerate searches because some *K*-mers are found less frequently than others and therefore result in fewer comparisons. In chromosome 1, the most common 4-mer (AAAA) appears 56 times more often than the rarest 4-mer (CGCG), and the most common 6-mer (TTTTTT) appears 929 times more often than the rarest 6-mer (CGTACG).

Each successive *K*-mer search is limited to the range of chunks that generated hits for the current working list. In the example above, after the CCCCGGGG hits have been identified, the search for AAAA is limited to the chunks between the first and last occurrence of CCCCGGGG. If a working list contains no hits, the search is terminated. This range limitation method can accelerate searches when a query has a small number of matches to the subject sequence.

### Searching with partially degenerate queries

A partially degenerate query can be expanded by searching for all of the possible matching sequences. For example, the restriction enzyme *Bsp*1286I has the recognition sequence GDGCHC, which can potentially match 9 nondegenerate sequences (when the second base is A, G, or T, and the fifth base is A, C, or T) and 40 partially degenerate sequences (when the second base is D, R, K, or W, or the fifth base is H, M, Y, or W). A search for GDGCHC will therefore return matches for all of the 49 possible matching 6-mers. Alternatively, searches for a partially degenerate query can be restricted to return only literal matches to that character string, so a search for "GDGCHC" will return matches only for the single 6-mer GDGCHC.

MICA scans a partially degenerate query to determine an efficient search strategy that limits the degeneracy of the constituent *K*-mers. For example, the restriction enzyme *Bae*I has the recognition sequence ACNNNNGTAYC, and for *K *= 4 the matches are found by searching for GTAY, TAYC, and ACNN. The MICA index is ordered lexicographically, so the *K*-mer ACNN invokes 16 contiguous disk reads from the Chunk Data Array, whereas the equivalent *K*-mers NNAC and NACN would invoke non-contiguous reads. Because contiguous reads are faster than non-contiguous reads, MICA pushes any degeneracy to the end of a *K*-mer whenever possible.

With a partially degenerate *K*-mer, the working list must be compared to multiple individual *K*-mer lists using an intersection algorithm. An obvious approach would be to adapt the method that is used with a single nondegenerate *K*-mer. In that case, MICA finds the intersection of the working list and the next *K*-mer list using a standard technique: a pointer is assigned to the *K*-mer list, and for each successive element in the working list, the pointer is advanced until the value in the *K*-mer list equals or exceeds the value in the working list [11]. When there are multiple *K*-mer lists, a pointer can be assigned to each one, and a working list element can be compared to all of the *K*-mer lists. However, this method becomes very inefficient if the working list is larger than the individual *K*-mer lists, because most of the comparisons fail to advance the pointers. MICA therefore uses an alternative intersection algorithm for partially degenerate *K*-mers. A boolean array of 65,535 elements is used to represent the positions in a chunk. For a given chunk, all of the individual *K*-mer lists are scanned, and the 2-byte position values are recorded by setting the corresponding boolean elements to true, yielding a boolean array that indicates which positions in the chunk match one of the *K*-mers. Then the intersection is obtained by checking whether each working list element corresponds to a value of true in the boolean array. This method is efficient due to the relatively small number of operations and the sequential nature of the memory accesses.

When a *K*-mer is very degenerate, a substantial amount of time may be needed to read and process the index data. In such cases, MICA switches to an alternate mode that uses the embedded DNA sequence. The entire query is compared to DNA sequence fragments that overlap each hit in the working list. Based on empirical tests, MICA was configured to perform this mode switch whenever the amount of index data that would need to be read exceeds 33% of the total DNA sequence data. This condition typically arises with extremely degenerate *K*-mers such as ANNN (*K *= 4) or ANNNNN (*K *= 6). Even with a less degenerate *K*-mer, the alternate mode is used if more data would be read by using the index than by directly examining the DNA sequence. Thus, at each stage of a search, MICA takes advantage of the fastest available option.

### Memory management during searches

Reading data from disk is slow, so the search speed can be maximized by pre-loading the entire file into main memory. MICA uses this approach when the sequence occupies up to 16 chunks. The corresponding files usually occupy no more than 3 MB of RAM and can be read from disk in a fraction of a second.

For longer sequences, as described above, MICA sacrifices some potential search speed in exchange for rapid index loading and low memory usage. The only parts of the index that are initially read into memory are the elements that describe the structure of the data arrays. During a search, the position values for the relevant *K*-mers are selectively read from disk. These read operations are usually the rate-limiting step in the search, but they are relatively efficient because of the compact 2-byte indexing format and because all of the positions for each *K*-mer are stored contiguously. Only a small portion of the index is used at a given time, so a typical search requires very little memory.

If a query sequence contains a degenerate or otherwise abundant *K*-mer, then reading the full list of position values for that *K*-mer might require more RAM than is available. MICA deals with this situation by dividing the subject sequence into segments and searching each segment in turn. During the search of a given segment, only the corresponding *K*-mer position values are read into memory.

## Results and discussion

MICA was coded in C++ and tested on a 2.5-GHz G5 Macintosh running OS X (10.4, Tiger) with 2.5GB of RAM. As subject data we used the May 2005 Ensembl release of the human genome [12], comprising 3.08 gigabases in 25 files representing the linear chromosomes 1–22, X, and Y, plus the circular mitochondrial chromosome. A simple graphical user interface was later constructed using Trolltech's Qt 4.1.

### Indexing performance

For a server application, a large index may be acceptable if sufficient memory is available, and slow indexing is acceptable because the index is created once and then used indefinitely. For a desktop application, smaller indexes are desirable because they occupy less disk space. Moreover, versatility is increased if the index can be created and updated rapidly, because this feature facilitates the analysis of new sequences and the modification of existing sequences.

Table 2 shows representative MICA index sizes and indexing times. With human chromosomes, storage requirements for the indexes were just under 2 bytes per base, reflecting the existence of N-stretches in the current genome assembly. With a computer-generated random sequence containing no degenerate base characters, the storage requirement was slightly over 2 bytes per base. The indexing time for chromosome 1, which is ~246 million bases, was 19.3 sec for *K *= 4 or 27.1 sec for *K *= 6. Only 56% of the *K *= 6 indexing time (15.3 sec) was due to the indexing procedure itself, with most of the remaining time being consumed by writing the completed index to disk. Indexing the entire human genome required 262 sec (4.4 min) for *K *= 4 or 345 sec (5.8 min) for *K *= 6. These speeds should enable a researcher to process a DNA database and move promptly to the analysis stage.

**Table 2:** Representative sizes, creation times, and loading times for MICA indexes

	*K*	Index Size (GB)	Index Creation Time (sec)	Index Loading Time (sec)
Chromosome 1 (2.46 × 10^8 ^bases)	4	0.42	19.3	0.023
	6	0.44	27.1	0.024
Random Sequence (2.46 × 10^8 ^bases)	4	0.46	23.5	0.020
	6	0.49	32.0	0.025
Human Genome (3.08 × 10^9 ^bases)	4	5.33	262	0.49
	6	5.67	345	0.44

The sequences of chromosome 1 and the 25 chromosomes comprising the human genome were obtained from the Ensembl database. We also tested a computer-generated random sequence of 246,000,000 bp containing equal proportions of G, A, T, and C. Index size refers to index elements E – M of the MICA file (see Table 1), and does not include the Sequence Segment. The creation time for each index includes the time needed to write the index to disk, but does not include the additional time needed to embed a copy of the sequence within the file; this additional embedding time for chromosome 1 was 16.4 sec for *K *= 4 or 19.6 sec for *K *= 6. Index loading refers to the reading of elements A – C and E – J of an index from disk into memory. For the human genome, the values listed are the sums of the values for the individual chromosomes.

To simulate indexing with limited RAM, we instructed MICA to index chromosome 1 using the procedure that would be followed if only 100 MB of RAM were available. The indexing time for *K *= 6 was 126 sec, which should still be adequate for most applications.

### Searching performance

The subject sequences were chromosome 1 or the entire human genome, and both DNA strands were searched. With the entire genome, the relevant index elements for each chromosome were loaded separately into memory for each search, but these loading times (Table 2) accounted for only a small fraction of the total search times. A series of searches was performed with nondegenerate queries of various lengths and with several partially degenerate queries.

Table 3 shows representative search times for *K *= 4. As expected, searches for 4-mers were the fastest, with each search requiring an average of 0.13 sec for chromosome 1 or 2.5 sec for the entire genome. Searches for 6- or 8-mers took about three times as long. For 15-mers, the average search times were 0.56 sec for chromosome 1 or 9.0 sec for the entire genome, about 50% longer than for 8-mers. As the query length increased further, the search times actually decreased as MICA took advantage of rare 4-mers within the queries.

**Table 3:** Representative search times for *K *= 4

Query	Chromosome 1		Human Genome	
	
	Time (sec)	Hits	Time (sec)	Hits
Nondegenerate 3-mers	0.82 [0.51]	6.96 × 10^6^	11.5	8.90 × 10^7^
Nondegenerate 4-mers	0.13 [0.028]	1.69 × 10^6^	2.5	2.17 × 10^7^
Nondegenerate 6-mers	0.35 [0.11]	160,702	5.8	2.05 × 10^6^
Nondegenerate 8-mers	0.38 [0.11]	16,631	6.2	213,099
Nondegenerate 15-mers	0.56 [0.11]	1.39	9.0	5.81
Nondegenerate 30-mers	0.54 [0.10]	1.03	8.3	1.24
Nondegenerate 100-mers	0.41 [0.069]	1.01	6.1	1.02
Nondegenerate 1000-mers	0.14 [0.019]	1.00	2.3	1.00
*Alu *30-mer fragment	0.77 [0.095]	1,130	13.6	14,041
GDGCHC (*Bsp*1286I)	0.43 [0.11]	398,999	6.9	4,776,086
GCCNNNNNGGC (*Bgl*I)	0.37 [0.12]	44,761	6.1	520,776
ACNNNNGTAYC (*Bae*I)	1.55 [0.34]	20,243	23.2	259,837

Both DNA strands were searched using *K *= 4. Results for the 3- to 1000-mer searches are average values obtained by searching with multiple queries. For 3-mers, all 64 possible nondegenerate queries were tested by extending each 3-mer to a partially degenerate 4-mer. For 4-mers, all 256 possible nondegenerate queries were tested. For 6- and 8-mers, 100 randomly chosen nondegenerate queries were tested. In the case of 15- to 1000-mers, each test involved 100 nondegenerate queries that were extracted randomly from chromosome 1 and checked to confirm that a given query had no more than 10 matches in the genome. The *Alu *30-mer fragment GGCCGGGCGCGGTGGCTCACGCCTGTAATC is a conserved sequence found at the 5' ends of *Alu *repeat elements [14]. The three partially degenerate queries are the recognition sequences for the restriction enzymes *Bsp*1286I, *Bgl*I, and *Bae*I. For chromosome 1, the search times without brackets were obtained after pre-loading only file elements A – C and E – J (see Table 1) into memory, and the faster search times with brackets were obtained after pre-loading the entire file. For the entire genome, the search times include the time needed to load elements A – C and E – J of each file into memory. Thus, the data for chromosome 1 reflect the time needed to search a file that is already open, whereas the data for the entire genome reflect the time needed to search a set of unopened files. To ensure that the search times without brackets reflect MICA performance for newly opened indexes, each search was preceded by a large number of extraneous reads, which flushed the main memory of any prior data from the relevant index.

Table 4 shows representative search times for *K *= 6. As expected, searches for 6-mers were the fastest. For 8- to 100-mers, the *K *= 6 searches were approximately five times as fast as the corresponding *K *= 4 searches. The reason for this difference is that read operations are the slowest step for most searches, and an average 6-mer occupies much less index space than an average 4-mer.

**Table 4:** Representative search times for *K *= 6

Query	Chromosome 1		Human Genome	
	
	Time (sec)	Hits	Time (sec)	Hits
Nondegenerate 3-mers	1.1 [0.80]	6.96 × 10^6^	14.8	8.90 × 10^7^
Nondegenerate 4-mers	0.28 [0.16]	1.69 × 10^6^	4.2	2.17 × 10^7^
Nondegenerate 6-mers	0.043 [0.0032]	160,702	0.96	2.05 × 10^6^
Nondegenerate 8-mers	0.079 [0.011]	16,631	1.6	213,099
Nondegenerate 15-mers	0.088 [0.0074]	1.39	1.8	5.81
Nondegenerate 30-mers	0.13 [0.0076]	1.03	1.8	1.24
Nondegenerate 100-mers	0.12 [0.0050]	1.01	1.7	1.02
Nondegenerate 1000-mers	0.094 [0.0023]	1.00	1.3	1.00
*Alu *30-mer fragment	0.12 [0.0055]	1,130	2.5	14,041
GDGCHC (*Bsp*1286I)	0.13 [0.031]	398,999	2.5	4,776,086
GCCNNNNNGGC (*Bgl*I)	0.44 [0.15]	44,761	6.2	520,776
ACNNNNGTAYC (*Bae*I)	1.40 [0.37]	20,243	21.1	259,837

Both DNA strands were searched using *K *= 6. The queries and the procedure were as described in Table 3, except that each 3- or 4-mer was extended to a partially degenerate 6-mer.

When the query length is less than *K*, the search times are relatively long because multiple *K*-mer lists must be merged. As an example for *K *= 4, the positions of each 3-mer were found by merging four 4-mer lists, so the 3-mer searches were much slower than the 4-mer searches (Table 3). For *K *= 6, the 4-mer searches were much slower than the 6-mer searches, and the 3-mer searches were slower still (Table 4). Multiway merges are naturally suited to parallel processing [13], and we are exploring the possibility of accelerating merges by harnessing the enhanced multithreading capacity of newer desktop computers.

For a given query length, searches are fast if there are very few matches to the query, and somewhat slower if multiple matches are distributed throughout the subject sequence. As a test case of a query with many matches, we used a conserved 30-base fragment of the *Alu *repeat element [14]. This *Alu *fragment was found 1,130 times in chromosome 1 and 14,041 times in the entire genome. The searches for the *Alu *fragment took only slightly longer than the searches for rare 30-mers (Tables 3 and 4). Thus, MICA delivers strong performance even with repeated sequences, which are challenging for some other search algorithms [5].

To test partially degenerate queries, we searched for the recognition sequences of the restriction enzymes *Bsp*1286I (GDGCHC), *Bgl*I (GCCNNNNNGGC), and *Bae*I (ACNNNNGTAYC). In the case of *Bgl*I, the search involved generating lists of positions for the 3-mers GCC and GGC, and then finding the intersection of those lists. As described above, 3-mer searches are expensive because of the merges, so MICA defers such merges until after the intersection operations. This approach made searching for the *Bgl*I recognition sequence about twice as fast as searching for a single 3-mer (Tables 3 and 4). The searches for the non-palindromic *Bae*I recognition sequence were relatively slow because MICA needed to process the data for the 2-mers AC and GT. In general, the most time-consuming searches are those involving *K*-mers with substantial degeneracy, because multiple individual *K*-mer lists need to be read from disk and then examined.

### Effects of varying *K*

We performed extensive tests with *K *chosen to be either 4 or 6. For *K*= 6 the individual *K*-mer reads were 16-fold smaller on average than for *K *= 4, yet the *K *= 6 searches for typical nondegenerate queries were faster by only about five-fold (Tables 3 and 4). The reason for this discrepancy is that the *K *= 6 reads are so small that disk seek times become limiting. Thus, increasing *K *to 7 would only marginally accelerate searches for typical nondegenerate queries. Moreover, a larger value of *K *would be detrimental with very short queries and with some partially degenerate queries. For example, when searching for the *Bgl*I recognition sequence, MICA expands the 3-mer GCC to the 4-fold degenerate GCCN for *K *= 4, but expands the same 3-mer to the 64-fold degenerate GCCNNN for *K *= 6 or the 256-fold degenerate GCCNNNN for *K *= 7. The best overall compromise for most purposes seems to be *K *= 6.

In the case of DNA molecules such as plasmids for which only a few KB are needed to store the DNA sequence, a *K *= 6 index is excessively large because it requires 24 KB to record how many times each nondegenerate *K*-mer is present. By contrast, a *K *= 4 index requires only 1.5 KB to store this information for a molecule of up to 65,535 bp. Therefore, MICA uses *K *= 4 if the DNA sequence fits within one chunk, or *K *= 6 if the DNA sequence occupies two or more chunks.

### Effects of memory usage

**Figure 2:**
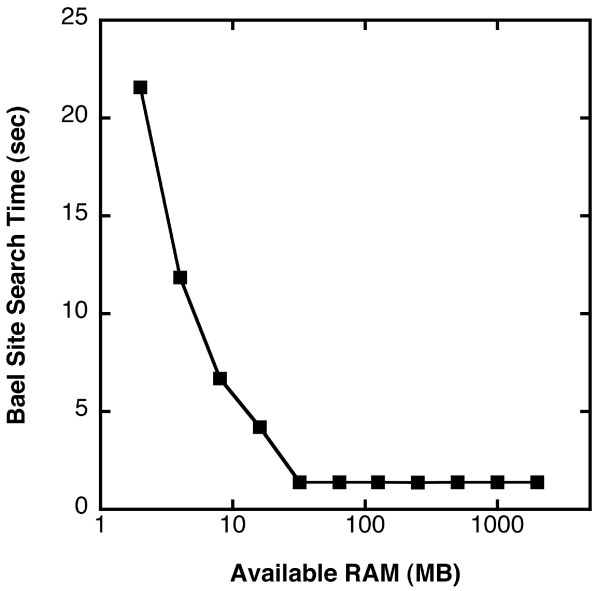
**Example of search times as a function of available RAM**. To simulate searching with various amounts of free memory, we instructed MICA (*K *= 6) to search chromosome 1 for *Bae*I sites (ACNNNNGTAYC) using the procedures that would be followed if the indicated amounts of RAM were available. For these measurements, each search was preceded by a large number of extraneous reads, which flushed the main memory of any prior data from the index.

For the genome-wide searches listed in Table 4, the amount of RAM used by MICA ranged from about 1.6 MB for a rare 6-mer to 40 MB for *Bae*I sites. These numbers are small because the searches were performed one chromosome at a time. To determine how memory limitation affects search times, we searched for *Bae*I sites in chromosome 1 under conditions that simulated different amounts of available RAM (Figure 2). The memory check algorithm estimated conservatively that searching all of chromosome 1 would require a maximum of 47.4 MB of RAM. As a result, when the available RAM dropped below this level, chromosome 1 was searched in segments. The search speeds decreased accordingly, but this decrease was not severe until the available RAM dropped below about 10 MB. Thus, even when a database contains large chromosomal sequence files, searching can be performed efficiently using a computer with modest amounts of memory.

Because all of the nuclear human chromosomes exceed 1 megabase in length, MICA does not load the full indexes into memory, but instead loads only the elements that describe the structure of the data arrays. The benefits of this strategy are rapid index loading and low memory usage. The disadvantage is that searches are slower than they would be if the full indexes were pre-loaded into memory. To quantify this effect, we repeated the test searches after pre-loading into memory the full MICA file for chromosome 1. The resulting values, listed in brackets in Tables 3 and 4, show that pre-loading accelerated the searches. This acceleration was dramatic with nondegenerate queries of length *K *or more. Yet the pre-loading operation took approximately 18 sec, a prohibitively long time if the goal is to do one or a few quick searches. By avoiding a time-consuming preparatory stage, MICA enables users to search large sequences without delay.

Immediately after indexing, the entire index is typically available in memory, so this time point is convenient for doing any routine searches. As a model for such a routine search, we indexed chromosome 1 using *K *= 6, and then searched for the recognition sequences of the 236 restriction enzymes sold by New England BioLabs. This search was fast at just under 11 sec.

### Relation to other approaches

The MICA index is hash-based but is actually related to suffix-based indexes. Hunt [15] has defined the "suffix sequoia" as a suffix tree derivative in which the entries are truncated at a string length of *K*. The result is a lexicographically-ordered *K*-mer array, which resembles the Chunk Data Array in the MICA index (Table 1), except that the suffix sequoia is about twice the size because absolute positions are stored as 4-byte integers. Another algorithm that resembles MICA is ACMES, which creates a hash table of 8-mers and accesses only the relevant hash bins during a search [16,17]. ACMES can find exact matches for queries of any length, although the indexes are large because this program was designed to search both sequence and annotation data. Thus, a number of researchers have converged on the same general strategy for indexing DNA sequences.

Despite these similarities, MICA has the following unusual features that make it advantageous, especially for desktop applications.

(1) A MICA index occupies only about 2*L *bytes, and a mammalian genome can be indexed in a few minutes. The previously described indexes that support comprehensive searching are either substantially larger or require much longer to generate.

(2) Rapid searching is accomplished without loading large amounts of data into main memory, because only a small fraction of each index is typically read from disk. Even if memory is quite limited, the search operations are still fast.

(3) Because MICA indexes 4- to 6-mers, very short queries can be matched quickly. This functionality is particularly useful for finding restriction sites. By comparison, ACMES indexes 8-mers and is therefore slower at matching very short queries [17].

(4) MICA can recognize and index all 15 characters of the standard DNA alphabet. By comparison, degenerate base characters are read as A's by SSAHA [5] and are excluded from the index by BLAT [6]. ACMES expands degenerate base characters by adding index entries for all of the corresponding nondegenerate 8-mers [17], but this approach has the disadvantage of indexing possible sequences as if they were actually present. With MICA, the index precisely captures the information in the original sequence, and the searches find all matches to any nondegenerate or partially degenerate query.

## Conclusion

MICA is designed to be a core indexing and search engine. Because the underlying approach is very simple, we were able to optimize the algorithms extensively to take advantage of the properties of modern desktop computers [18]. The end result meets our goal of enabling users to open and search large DNA databases rapidly on computers with limited RAM.

In its present form, MICA is ideally suited to comprehensive searching for exact matches in a DNA database. Such a database might represent, e.g., a genome or a collection of plasmid vectors. Potential applications include: *in silico *restriction enzyme digestion, which can be used to type organisms by amplified fragment length polymorphism (AFLP) analysis or pulsed field gel electrophoresis [19,20]; "virtual PCR" to predict the specificity of PCR amplification from complex templates [21,22]; and the automated definition of oligonucleotide-flanked sequence-tagged sites (STSs) in genomic sequences [23,24]. We are incorporating MICA into desktop software that allows for versatile browsing and manipulation of chromosome-sized DNA sequences. For example, a MICA-based PCR simulator allows us to simulate a PCR amplification from human genomic DNA in 2–3 sec.

MICA could be extended by adding alignment algorithms for identifying sequences that are similar but not identical to the query. Such algorithms have been widely studied and implemented [1,7-9,15,25,26], and they can benefit greatly from using an index to find "seeds" for the alignments [6,27,28]. In addition, MICA could easily be modified to operate in server mode. For this purpose, faster searching of large sequences would be achieved by loading the complete indexes into memory, as illustrated in Tables 3 and 4.

## Availability and requirements

**Project name: **MICA – Desktop Software for Comprehensive Searching of DNA Databases

**Project home page: **MICA binaries for Macintosh OS X (mica_mac.dmg) and Windows (mica_1.0_win.exe) are available at .

**Operating systems: **Macintosh OS X (10.3.9 or higher) and Windows (NT, 2000, XP)

**Programming language: **C++

**Other requirements: **None

**License: **Freely available for academic and non-profit use. Researchers can request assistance with obtaining the MICA source code and integrating it with other software.

**Any restrictions to use by non-academics: **Commercial users require a license. For questions regarding commercial uses, please contact the University of Chicago's Office of Technology and Intellectual Property, UCTech, at (773) 702–1692 or www.uctech.uchicago.edu.

## Authors' contributions

WAS generated the code, and BSG guided the project. Both authors contributed to the algorithm design.

Both authors read and approved the final manuscript.
